# Identification and characterization of the ‘missing’ terminal enzyme for siroheme biosynthesis in α-proteobacteria

**DOI:** 10.1111/mmi.12542

**Published:** 2014-03-13

**Authors:** Shilpa Bali, Sarah Rollauer, Pietro Roversi, Evelyne Raux-Deery, Susan M Lea, Martin J Warren, Stuart J Ferguson

**Affiliations:** 1 Department of Biochemistry, University of OxfordSouth Parks Road, Oxford, OX1 3QU, UK; 2 Sir William Dunn School of Pathology, University of OxfordSouth Parks Road, Oxford, OX1 3RE, UK; 3 School of Biosciences, University of KentCanterbury, Kent, CT2 7NJ, UK

## Abstract

It has recently been shown that the biosynthetic route for both the *d*_1_-haem cofactor of dissimilatory *cd*_1_ nitrite reductases and haem, via the novel alternative-haem-synthesis pathway, involves siroheme as an intermediate, which was previously thought to occur only as a cofactor in assimilatory sulphite/nitrite reductases. In many denitrifiers (which require *d*_1_-haem), the pathway to make siroheme remained to be identified. Here we identify and characterize a sirohydrochlorin–ferrochelatase from *Paracoccus pantotrophus* that catalyses the last step of siroheme synthesis. It is encoded by a gene annotated as *cbiX* that was previously assumed to be encoding a cobaltochelatase, acting on sirohydrochlorin. Expressing this chelatase from a plasmid restored the wild-type phenotype of an *Escherichia coli* mutant-strain lacking sirohydrochlorin–ferrochelatase activity, showing that this chelatase can act in the *in vivo* siroheme synthesis. A Δ*cbiX* mutant in *P. denitrificans* was unable to respire anaerobically on nitrate, proving the role of siroheme as a precursor to another cofactor. We report the 1.9 Å crystal structure of this ferrochelatase. *In vivo* analysis of single amino acid variants of this chelatase suggests that two histidines, His127 and His187, are essential for siroheme synthesis. This CbiX can generally be identified in α-proteobacteria as the terminal enzyme of siroheme biosynthesis.

## Introduction

**Fig. 1:**
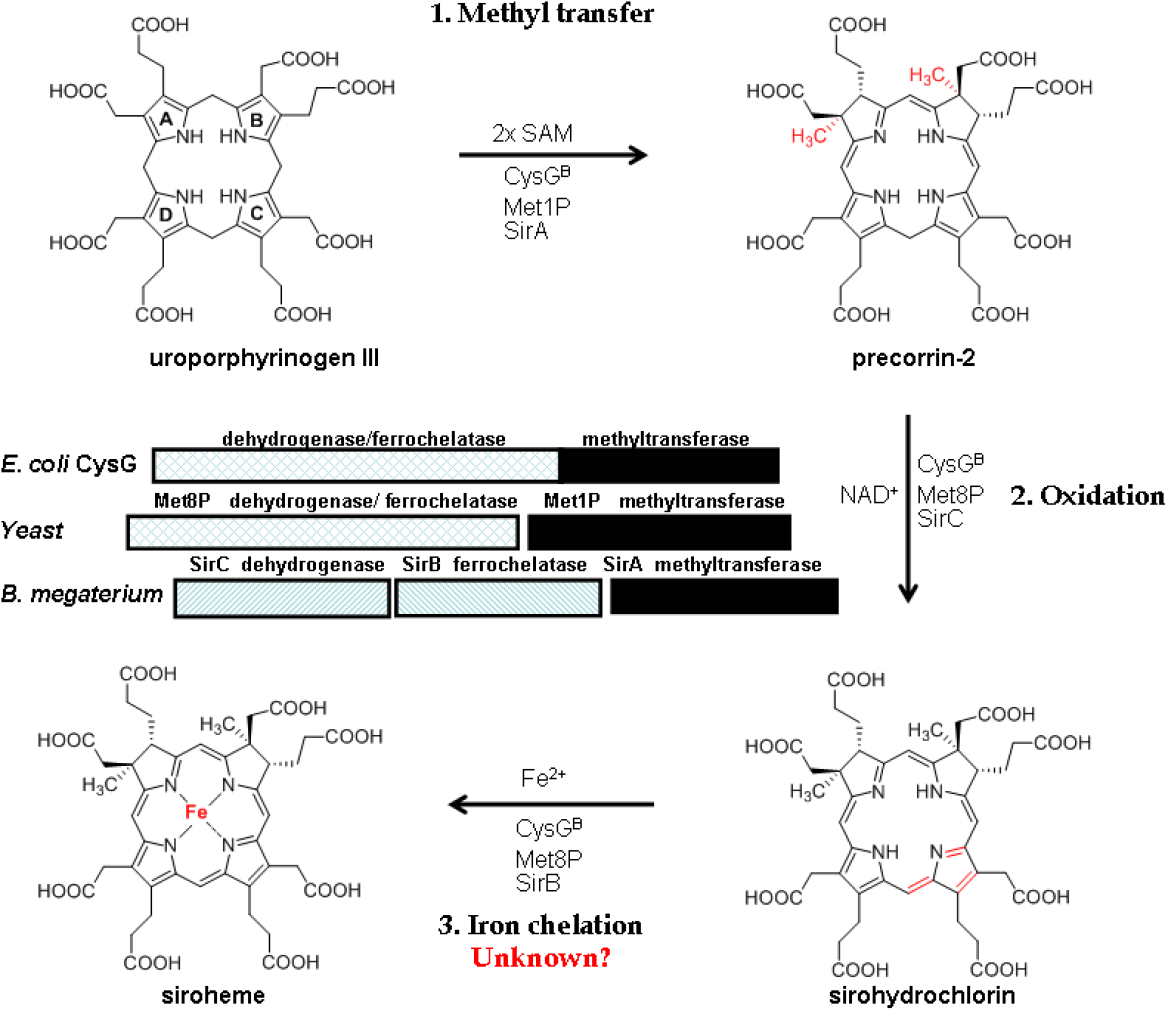
Biosynthesis of siroheme. Siroheme is synthesized from uroporphyrinogen III in three consecutive enzymatic steps *via* the intermediates precorrin-2 and sirohydrochlorin. In *E. coli* the SAM-dependent methylation of uroporphyrinogen III, the NAD^+^ dependent dehydrogenation of precorrin-2 (PC-2) and the iron insertion into sirohydrochlorin is catalysed by the multifunctional enzyme CysG. In yeast the methyl transfer is catalysed by Met1p and the oxidation of (PC-2) and iron chelation of sirohydrochlorin is catalysed by the bifunctional enzyme Met8p, whereas in *B. megaterium* there are three monofunctional enzymes SirA, SirB and SirC for siroheme synthesis. Notably, in many prokaryotes, CysG^A^, Met8p and SirB like proteins are missing even though these organisms still require siroheme for assimilatory pathways.

Siroheme is a known prosthetic group for assimilatory sulphite and nitrite reductases that carry out the six electron reduction of sulphite to sulphide and nitrite to ammonia respectively (Swamy *et al*., 2005; Tripathy *et al*., 2010; Fischer *et al*., 2012). Apart from its role in assimilation of sulphur and nitrogen in a variety of organisms, siroheme is also associated with the dissimilation of sulphite-to-hydrogen sulphide in dissimilatory sulphite reductases (dSiR) (Simon & Kroneck, 2013), where this cofactor is also coupled to a [4Fe-4S] centre. The biosynthesis of the tetrapyrrole-derivative siroheme represents the shortest tetrapyrrole biosynthesis pathway, as it comprises only three chemical transformations, starting from the universal tetrapyrrole precursor uroporphyrinogen III (uro'gen III). The first of these transformations is catalysed by a SAM (S-adenosyl-l-methionine) dependent uroporphyrinogen III methyltransferase (SUMT), which converts (uro'gen III) to precorrin-2 (PC-2). The second step involves the dehydrogenation of precorrin-2 to sirohydrochlorin by a NAD^+^-dependent precorrin-2 dehydrogenase. The final step involves the insertion of iron into sirohydrochlorin and is catalysed by a sirohydrochlorin ferrochelatases (Warren *et al*., 1994). In *E. coli* and *Salmonella enterica* siroheme is known to be synthesized by CysG, which is a multifunctional enzyme that carries all three enzymatic activities on a single polypeptide that forms a homodimeric protein (Warren *et al*., 1994; Stroupe *et al*., 2003). However, other organisms make siroheme *via* considerably different enzyme complements, and in some cases unidentified enzymes must be involved. For example, in *Saccharomyces cerevisiae*, there are two different enzymes: Met1p, which catalyses the formation of PC-2 from uro'gen III, and Met8p a bi-functional dehydrogenase and a ferrochelatase (Hansen *et al*., 1997; Raux *et al*., 1999; 2003). On the other hand, in *Bacillus megaterium*, there are three separate enzymes: the SAM-dependent methyltransferase encoded by *sirA*, an NAD^+^ dependent PC-2 dehydrogenase, encoded by *sirC* and a sirohydrochlorin ferrochelatase encoded by *sirB* (Fig. 1) (Leech *et al*., 2002; Brindley *et al*., 2003; Schubert *et al*., 2008).

**Fig. 2:**
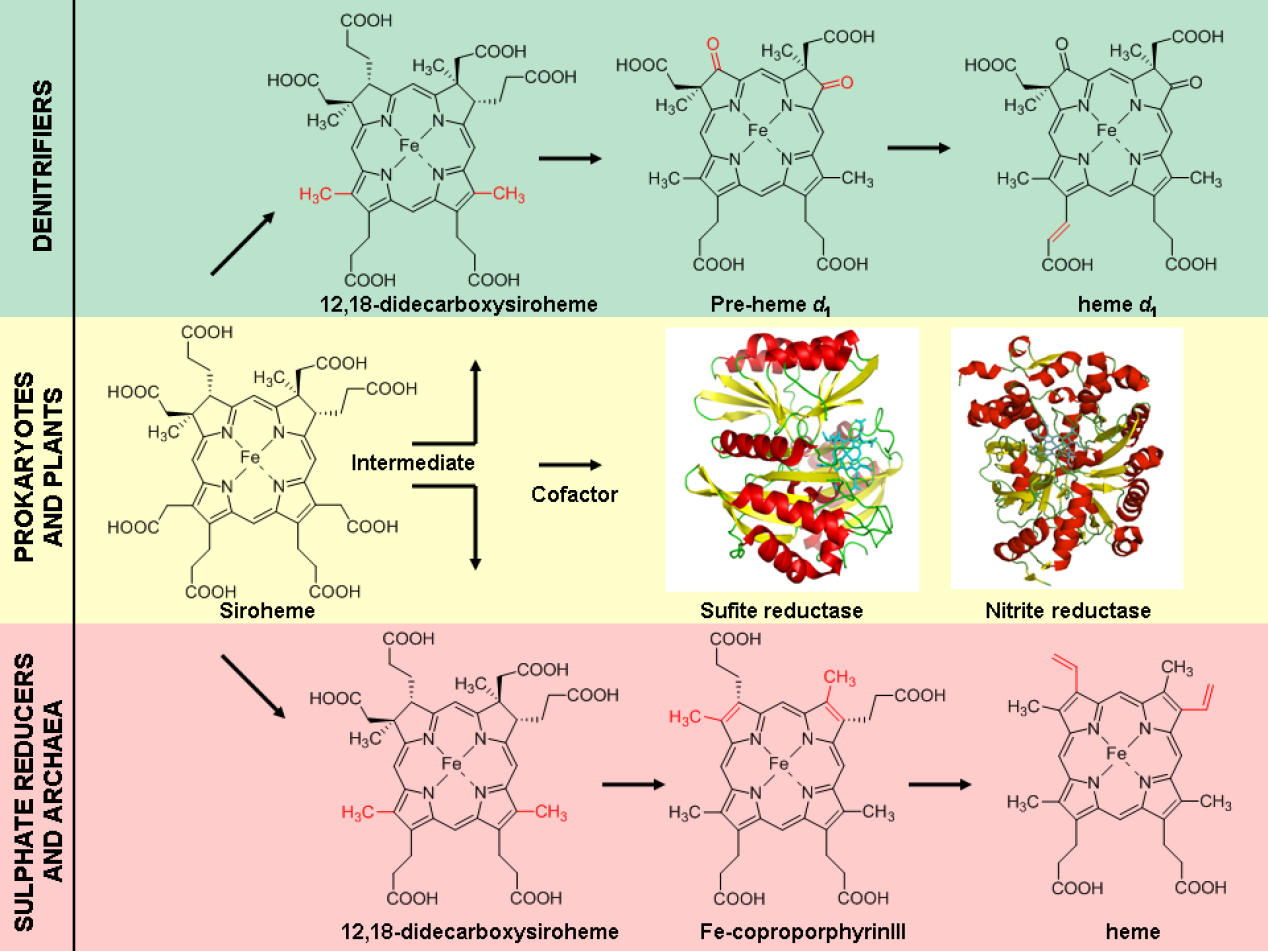
Siroheme as a branch point during tetrapyrrole metabolism. Siroheme is used as a prosthetic group in assimilatory sulphite and nitrite reductases in plants and various prokaryotes. However, it is also an intermediate during the biosynthesis of haem *d*_1_, which is a unique cofactor of dissimilatory cytochrome *cd*_1_ nitrite reductases in various denitrifying bacteria. Most of the archaea and sulphate reducing bacteria are now also known to use siroheme as a precursor for making haem *via* a novel route, so siroheme represents another branch point during tetrapyrrole metabolism after uroporphyrinogen III that can be used for making tetrapyrroles such as haem, chlorophyll and cobalamin. PDB accession code for sulphite reductase is 2AOP and for nitrite reductase is 2AKJ.

Siroheme is both an end-product and an intermediate in tetrapyrrole metabolism. It can not only act as a cofactor in assimilatory sulphite and nitrite reductases in many organisms, such as eubacteria, archaea, plant and fungi (Tripathy *et al*., 2010) but unexpectedly siroheme was also shown to act as a key intermediate during the biosynthesis of *d*_1_ haem in denitrifying bacteria, and in the alternative route of making haem within many archaea and sulphate reducing bacteria (Bali *et al*., 2011) (Fig. 2). Multiple genome analyses of organisms that use siroheme in d1 synthesis have shown that the characteristic siroheme synthesis proteins such as CysG, Met1p and Met8p are often absent. Therefore, it is important to investigate and understand how siroheme is synthesized in organisms that have an overlapping requirement for this small molecule as both a cofactor and an intermediate in a tetrapyrrole biosynthesis pathway. For example, two closely related denitrifying bacteria, *Paracoccus denitrificans* and *Paracoccus pantotrophus*, and other denitrifiers (mostly belonging to the α-proteobacteria class), lack the known enzymes of siroheme synthesis pathway. Apart from biosynthesis, the possible regulation of the siroheme synthesis pathway in such organisms is also intriguing as siroheme is needed for denitrification, an anaerobic process, but also for nitrogen and sulphur assimilation, a process that is often associated with aerobic metabolism.

In the *P. denitrificans* genome there are at least two separate genes that encode SAM dependent uro'gen III methyltransferases (SUMTs), which are associated with the first step of siroheme biosynthesis. One of these SUMTs, encoded by Pden_2488, is found in the beginning of the *nir* operon, which codes for enzymes involved in the biosynthesis of *d*_1_ haem, and is termed *nirE* in this operon. The second methyltransferase is encoded by Pden_2556 and is found close to the vitamin B_12_ biosynthesis operon. Extensive *in vivo* and *in vitro* analysis of NirE from *P. pantotrophus* and *Pseudomonas aeruginosa* has shown its role in the formation of precorrin-2 from uro'gen III (Storbeck *et al*., 2009; 2011; Zajicek *et al*., 2009). There are no obvious candidates for the PC-2 dehydrogenase enzyme on the genome that could generate sirohydrochlorin from PC-2 or for a ferrochelatase that could catalyse siroheme production from sirohydrochlorin. Intriguingly, there is a gene (Pdne_2332), which annotates as *cbiX* on the *P. denitrificans* genome. Generally, CbiX catalyses the chelation of cobalt into sirohydrochlorin, an early step associated with the anaerobic biosynthesis of vitamin B_12_ (cobalamin) found in various microorganisms (Moore and Warren, 2012). However, in *P. denitrificans*, this protein is unlikely to be a cobaltochelatase, since in this organism an aerobic route of making cobalamin operates and it requires exogenous vitamin-B_12_ during anaerobic growth for the expression of a cobalamin-dependent ribonucleotide reductase, which is essential during anaerobic growth conditions (Shearer *et al*., 1999). In addition, the genome of *P. denitrificans* has another gene (Pdne_2532) that annotates as *cobN*, which encodes a cobaltochelatase for aerobic Vitamin-B_12_/Cobalamin synthesis and it is present in the gene cluster that has several other genes that are involved in the aerobic biosynthesis of cobalamin. An important observation we made was that some denitrifying bacteria, such as *Roseobacter denitrificans*, *Polymorphum gilvum* and *Dinoroseobactor shibae*, have a putative *cbiX* located after *nirN*, which is at the end of their *nir* operons that are associated with *d*_1_ haem biosynthesis *via* siroheme. Taking these observations together led us to the hypothesis that the CbiX protein from *P. pantotrophus* might be the chelatase for making siroheme. To this end we have used a range of methods to establish its physiological function in *Paracoccus* and obtained its crystal structure.

## Results

**Fig. 3:**
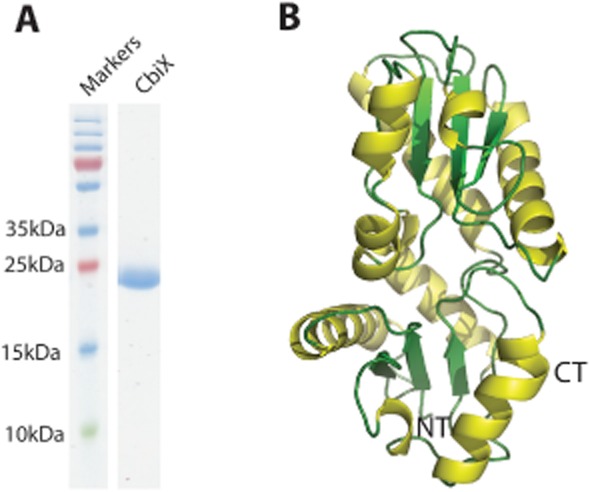
Structural characterization of CbiX.A. SDS-PAGE showing the final purified sample of CbiX.B. The structure of CbiX, helices shown in yellow, β-strands shown in green and loops coloured in light green. NT is N terminus and CT is C terminus.

Our previous work on *d*_1_ haem biosynthesis was performed in *P. pantotrophus* (Bali *et al*., 2011) and so we chose to clone and express the gene annotated as *cbiX* from that organism. CbiX from *P. pantotrophus* (Pp-CbiX) was cloned into an *E. coli* expression vector and was purified (Fig. 3A) as a homogenous, monomeric protein, as shown by multi angle laser light scattering with a yield of 20 mg protein l^−1^ of growth culture. In addition, with this purified sample, crystals were grown and the crystal structure of Pp-CbiX was determined to a resolution of 1.9 Å (Fig. 3B).

### Sirohydrochlorin ferrochelatase activity of CbiX and its variants

To determine whether the Pp-CbiX WT enzyme is a ferrochelatase or a cobaltochelatase, the catalytic activities of the expressed CbiX and its variants were investigated *in vivo* by testing for complementation of an *E. coli* Δ*cysG* mutant derivative strain (Cys247/Table 1). The strain Cys247 is unable to grow on M9 minimal medium without cysteine supplementation because although the *cysG* mutant derivative strain of *E. coli* harbours a plasmid pCIQ-*cobA*, which encodes genes expressing SUMT from *Ps. denitrificans,* it lacks the bifunctional chelatase and dehydrogenase activity. Therefore, the phenotype can only be rescued if the cells are transformed with a plasmid carrying a gene that encodes a ferrochelatase, which would restore siroheme production in the cells. Simple complementation of the Cys247 strain with plasmid based Pp-CbiX gave colonies on minimal medium agar plates that lacked cysteine, showing that this CbiX could restore the siroheme production in the Cys247 strain. Exogenous cobalt (CoCl_2_.6H_2_O) was added in order to determine the specificity of Pp-CbiX for Fe^2+^ *versus* Co^2+^. The results in Table 1 suggest that Pp-CbiX is a ferrochelatase, as when Cys247::*cbiX_Pp* cells were plated on M9 medium agar with an increasing concentration of cobalt there was no detrimental effect on the growth of cells in the absence of cysteine. This shows that Pp-CbiX could still use the limited amount of iron present in the growth medium in preference to the exogenously added Co^2+^ in order to synthesize siroheme. In contrast, when Cys247::*cbiK_St* cells (where CbiK is known to insert cobalt or iron into sirohydrochlorin) were plated on M9 medium agar in absence of cysteine and with increasing concentration of cobalt (from 5 μM to 30 μM CoCl_2_), the cells stopped growing (see supplemental material), consistent with CbiK having a preference for cobalt over iron as it is a known cobaltochelatase of the anaerobic cobalamin biosynthesis pathway (Raux *et al*., 1997).

**Table 1:** Results from *in vivo* ferrochelatase activity tests

Protein	M9 medium+ l-cysteine	M9 medium+ No l-cysteine	M9 medium+ Co^2+^
WT Pp-CbiX	+++	+++	+++
Pp-CbiX^H10A^	+++	+++	++
Pp-CbiX^Q12A^	+++	+++	++
Pp-CbiX^H127A^	+++	—	—
Pp-CbiX^H187A^	+++	—	—
Pp-CbiX^D190A^	+++	+++	++
Pp-CbiX^D191A^	+++	+++	++
Pp-CbiX^E126A^	+++	+++	++
*E. coli*-CysG	+++	+++	+++
Se-CbiK	+++	+++	—
Expression vector only	++	—	—

The relative extent of growth of Δ*cysG E. coli* (Cys247) strain complemented with *Paracoccus pantotrophus* CbiX or its variants on M9 minimal medium was estimated by eye and is indicated by the number of plus signs. Cobalt is used at 1 μM of medium and l-cysteine is used at 50 μg l^−1^ of medium. Growth curves for representative strains are presented in Fig. S5.

The catalytic activities of Pp-CbiX mutant variants were then investigated *in vivo* by testing whether they were also able to complement the *E. coli* Cys247 strain. In most cases (see Table 1), *E. coli* Cys247 strains carrying pET14b derivative plasmids were able to grow on minimal medium, indicating that the CbiX variants were active *in vivo*. However, those cells complemented by the CbiX^H127A^ or CbiX^H187A^ mutants did not grow on M9 medium agar plates unless supplemented with exogenous cysteine, indicating that they lacked the ferrochelatase activity and the lack of the complementation of these two strains was not due to the lack of CbiX-H127A and -H187A mutant proteins in the cell. Thus, (His127) and (His187) were shown to be important for the *in vivo* chelatase activity of Pp-CbiX (Table 1). These histidines are equivalent to the metal ion and/or porphyrin binding residues in the family of ATP-independent type II chelatases (Raux *et al*., 2003) to which Pp-CbiX belongs on the basis of sequence analysis.

### Unmarked gene deletion of *P. denitrificans* *cbiX*

In order to determine the *in vivo* function of this putative chelatase in a denitrifier, an unmarked gene deletion strain, (SBN69) in of Pdne_2332, ORF was constructed in *P. denitrificans* (an organism with a sequenced genome and methods available to obtain gene knockouts). This chelatase deficient strain failed to grow anaerobically on a minimal medium supplemented with nitrate (as terminal electron acceptor) and accumulated nitrite. This result can be interpreted as the direct consequence of the loss of ability of this strain to make siroheme *de novo* and hence to reduce nitrite, owing to a failure to synthesise *d*_1_ haem that is required for the assembly of *holo*-cytochrome *cd*_1_ nitrite reductase. However, when *cbiX* was expressed from the plasmid (pSB131) in this unmarked deletion strain, the resultant strain (SBN70) could denitrify. In addition, the aerobic growth of SBN69 on minimal medium agar plates required supplementation with NH_4_^+^ and cysteine. This result again suggested that the deletion strain was incapable of synthesizing siroheme for assimilation of sulphur and nitrogen. Furthermore, the SBN70 strain could grow on minimal medium plates without exogenous addition of cysteine or NH_4_^+^ showing that the plasmid based CbiX could complement the deletion mutant strain for aerobic assimilation of sulphur and nitrogen.

### The structure of Pp-CbiX

**Fig. 4:**
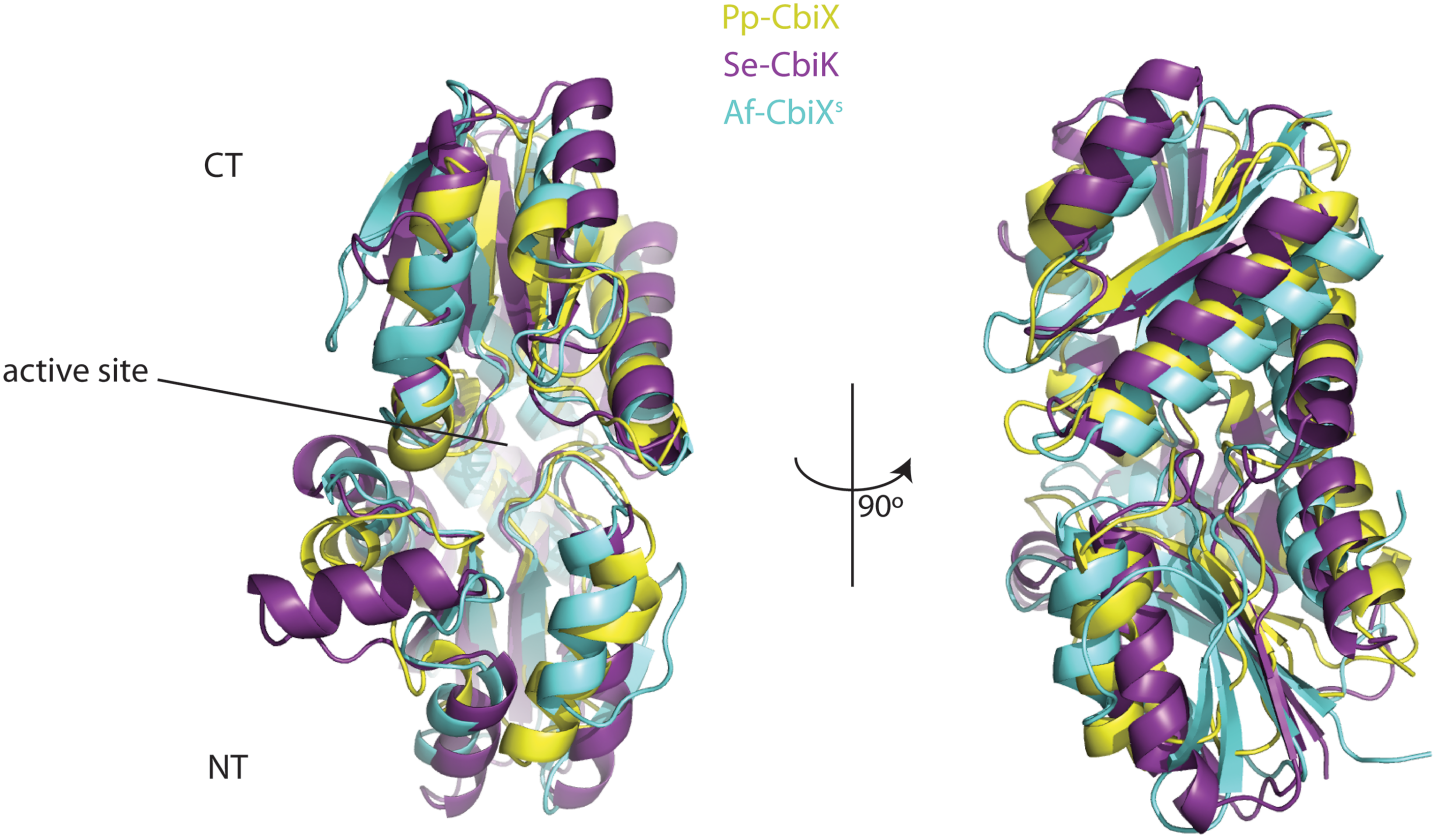
Structural comparison of CbiX with CbiX^S^ and CbiK. An overlay of Pp-CbiX (in yellow) with the archaeal cobaltochelatase Af-CbiX^S^ (in cyan) and with the bacterial cobaltochelatase Se-CbiK (in purple) is shown in different colours. NT and CT are N-terminus and C-Terminus respectively.

The overall structure (Fig. 3B) of Pp-CbiX is characteristic of the type II class of metal chelatase enzymes. A search of the PBD using PDBeFold (EMBL-EBI) showed that the full length Pp-CbiX protein is most similar to CbiK from *Salmonella enterica*, a cobaltochelatase (Se-CbiK). These two proteins overlay with a root mean square deviation (RMSD) of 3.13 Å (Fig. 4) despite a low sequence identity of 20% (ClustalW2). Given that the type II chelatase family has a two-domain architecture with varying degrees of tilt seen between the two domains, the Pp-CbiX structure was split into a N-terminal (NT) and C-terminal (CT) domain to identify structures similar to each domain in turn. Splitting the structure in this way identified the most similar structure to the Pp-CT domain as the CbiX^S^ protein from *Archaeoglobus fulgidus* (Af-CbiX^s^, with a metallated sirohydrochlorin bound), with an RMSD of 1.88 Å. Af-CbiX^S^ has been regarded as a primitive cobaltochelatase; it is half the size of the other known class II chelatases and acts as a symmetrical dimer to carry out the insertion of cobalt into sirohydrochlorin (Yin *et al*., 2006). It is believed that the type II chelatase enzymes may have evolved into asymmetrical two-domain proteins from dimers of these smaller precursor proteins (Brindley *et al*., 2003). Finally, the NT domain of Pp-CbiX was most similar to the SAV_2001 protein from *Streptomyces avermitilis*, a protein with unknown function (RMSD 2.69 Å).

### Catalytically important residues in Pp-CbiX

**Fig. 5:**
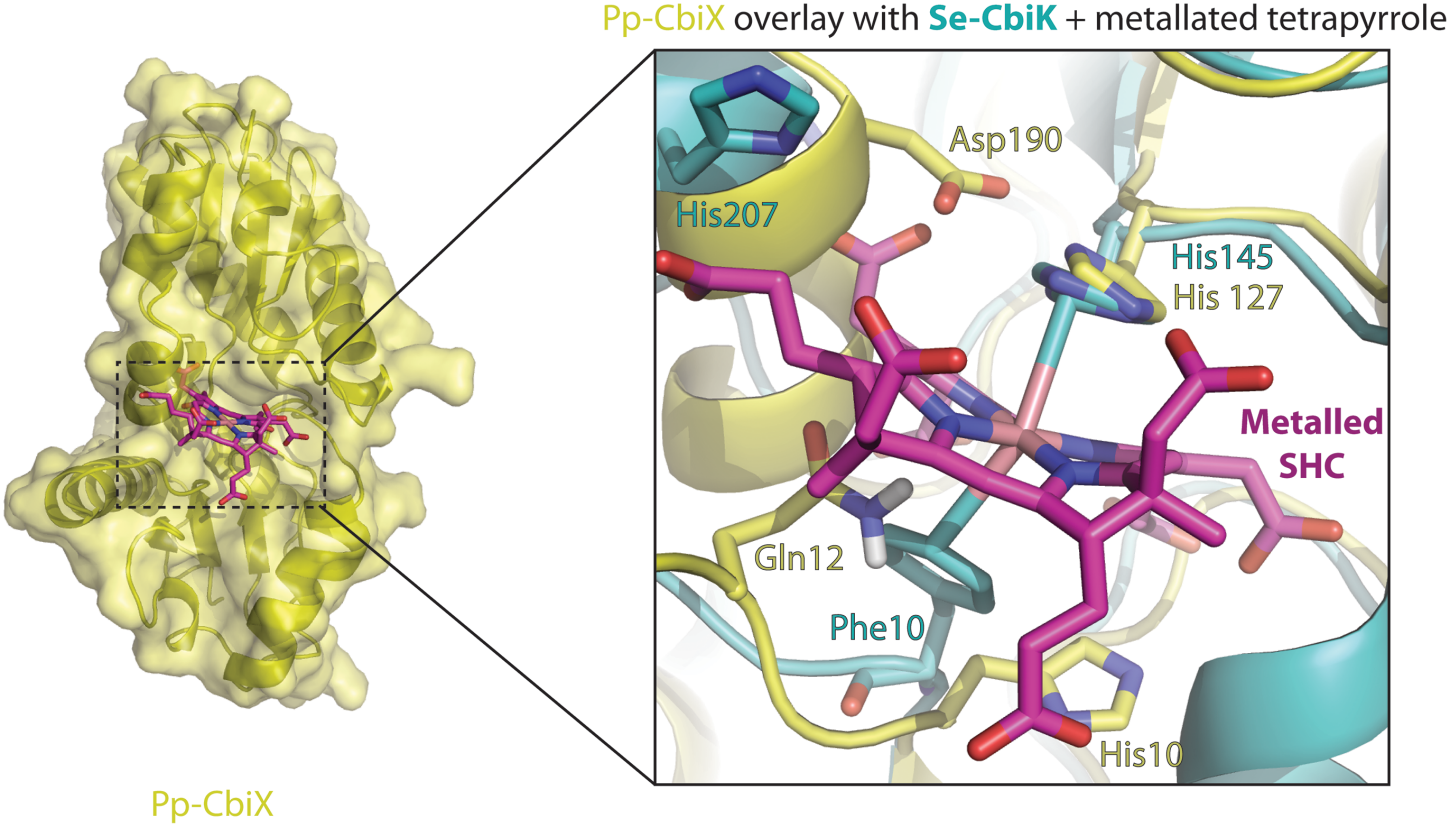
Investigation of the active site of CbiX.A. A ribbon and surface representation of CbiX, showing a metallated sirohydrochlorin in the active site, based on the co-crystal structure from Se-CbiK.B. A zoomed in view of the active site, Pp-CbiX shown in yellow and Se-CbiK in cyan. The metallated sirohydrochlorin is shown in pink. Residues important in CbiK function and structurally equivalent CbiX residues are shown as sticks. Helix 7, which contains a second residue important for metal ion binding (i.e. His207) in the Se-CbiK (purple), is shown with the product analogue, overlaid with Pp-CbiX (yellow).

To understand how Pp-CbiX may act as a ferrochelatase, the structure of Se-CbiK in complex with metallated sirohydrochlorin was used for a comparison, given that the Se-CbiK has the most similar overall architecture to Pp-CbiX and both these enzymes insert a metal ion (either iron or cobalt) into sirohydrochlorin. The structural overlay shows the metallated tetrapyrrole fits into the central cavity in Pp-CbiX (Fig. 5) and indicates that the active site residues in Pp-CbiX are located in the CT half of the protein, similar to Se-CbiK but unlike CbiX^L^ and SirB that have active site residues in their NT (Fig. 5). The overlay highlights the conserved position in the active site of the (His127) residue of Pp-CbiX with the equivalent His residue in Se-CbiK (His145), shown to be involved in metal binding in the bound tetrapyrrole. The functional importance of (His127) from Pp-CbiX was demonstrated by mutating this residue to alanine, which resulted in the loss of *in vivo* ferrochelatase activity of Pp-CbiX^H127A^ (Table 1).

The structure of Se-CbiK in complex with metallated sirohydrochlorin previously showed how a second residue, (Phe10), was adjacent to the metal within the centre of the tetrapyrrole ring (Romao *et al*., 2011). In comparison, the structure of Pp-CbiX revealed two candidate residues at this region of the structure that may act in a similar role, (Gln12) and (His10). However, switching both of these residues to alanine did not have an effect on the *in vivo* ferrochelatase activity of Pp-CbiX, showing that despite their appropriate position within the structure, these residues are not significantly involved in metal insertion.

The crystal structures of the *Bacillus subtilis* ferrochelatase (Bs-HemH) in complex with various metal ions, and a periplasmic cobaltochelatase from *Desulfovibrio vulgaris* (Dv-CbiK^P^) in complex with cobalt, were used to identify and test the equivalent amino acid residue(s) in Pp-CbiX, which might be important in metal ion binding before the metal ion insertion into the tetrapyrrole substrate (Hansson & Hederstedt, 1994, Hansson *et al*., 2007, Hansson *et al*., 2011, Romao *et al*., 2011). The co-crystal structures from the cobaltochelatase Dv-CbiK^P^-Co^2+^ showed that a histidine residue (His207) was responsible for binding the free metal ion, whereas a glutamate (Glu264) residue was shown, both structurally and functionally, to be used by the ferrochelatase Bs-HemH. It was speculated that either Asp190 or Asp191 of Pp-CbiX may play an equivalent role to Glu264 in Bs-HemH (Fig. 5), may be involved in the specific binding of iron in the active site of Pp-CbiX in preference to cobalt. However, mutagenesis of these residues to alanine in Pp-CbiX resulted in active sirohydrochlorin ferrochelatase showing that they are not essential for function. The nearby (Glu186) was also shown to be non-essential by the *in vivo* functional assays (Table 1). Given the structural comparison to the ferrochelatase was unsuccessful in identifying the Pp-CbiX residue(s) that carries out the metal binding role, multiple sequence alignments were performed to gain insight into which residues are highly conserved and thus plausibly important for Pp-CbiX function. The alignments revealed the equivalence of a functionally essential His residue in both Se-CbiK and Af-CbiX^S^ with (His187) of Pp-CbiX. Mutagenesis of (His187) in our protein demonstrated that this residue was indeed essential for the ferrochelatase activity of Pp-CbiX (Table 1). The putative use of a histidine residue for the metal binding role, in contrast to a glutamate residue seen in other type II ferrochelatases, makes Pp-CbiX unusual.

Interestingly, in the structure of Pp-CbiX, the functionally essential (His187) points away from the active site of the enzyme. Our structure indicates that in order for (His187) to perform its role, a large scale movement of this region of the Pp-CbiX structure must occur, so that (His187) could point into the position of the metal within the active site. It is interesting to note that such a large scale movement of the equivalent His residue in the cobaltochelatase Se-CbiK (His207), has already been noted (Romao *et al*., 2011). Comparison between the *apo* structure of Se-CbiK and the Se-CbiK bound metallated-SHC structure revealed how Se-CbiK, (His207) undergoes a relatively large, 5.5 Å, movement of its β carbon atom to allow the His side-chain to move out of the way of the tetrapyrrole (Romao *et al*., 2011). So it is feasible that (His187) of Pp-CbiX is also at a very flexible region of the structure, and this crystal form has captured the (His187) residue in an inactive conformation in which it is moved out of the way of the active site.

The structure of Pp-CbiX is most similar in both its overall 3D structure, as indicated by RMSD, and in terms of the active site residues it uses, to the type II cobaltochelatases. However, our *in vivo* functional data (Table 1) has demonstrated that Pp-CbiX acts as a ferrochelatase, by acting to insert iron into the tetrapyrrole substrate in preference to cobalt ion.

## Discussion

The importance of siroheme has increased markedly because of our recent discovery that it is an intermediate on pathways to *d*_1_ haem and *b*-haem synthesis in a range of organisms (Bali *et al*., 2011). In this context, it is important to understand how siroheme is synthesized in an organism that utilizes one of these overlapping pathways for tetrapyrrole metabolism. Here we have confirmed our hypothesis that *cbiX*, sometimes but not always found adjacent to *nir* operon, is not primarily a cobaltochelatase but rather the missing terminal enzyme of siroheme synthesis in many denitrifying bacteria. The structure of Pp-CbiX was determined to 1.9 Å resolution and compared with the structures of other known type II chelatases. Various structures of cobaltochelatases that are associated with the anaerobic cobalamin biosynthesis (for example CbiK from *S. enterica*, CbiX^S^ from *A. fulgidus* and CbiX from *D. vulgaris*) are available (Yin *et al*., 2006; Romao *et al*., 2011) but no structure of sirohydrochlorin ferrochelatase (SirB) is reported so far. Our protein, Pp-CbiX represents the first sirohydrochlorin ferrochelatase structure. The structure of Pp-CbiX reveals that despite its low sequence conservation to the type II chelatases, it has the characteristic fold and the active site seen in this class. However, the *apo*-Pp-CbiX structure alone cannot give clues to the reason for the metal ion specificity of Pp-CbiX. Therefore, further structures of Pp-CbiX in complex with either sirohydrochlorin or iron are required to elucidate the molecular basis for its metal ion specificity. Despite extensive attempts to acquire both of these structures through co-crystallisation these attempts were unsuccessful. Therefore, in an attempt to try and identify the key residues in this enzyme, the structure was compared with those of other type II chelatases. Substrate recognition and metal ion binding was tested in our ferrochelatase by utilizing the *in vivo* growth analysis in *E. coli*. Pp-CbiX has highly conserved histidine residues in its sequence (Fig. S1). We found that both (His127) and (His187) of Pp-CbiX were essential for the chelatase function. These two histidines must be contributing to the role of metal ion and substrate binding in our ferrochelatase. Alternatively, histidine is also known to be capable of acting as a Lewis base, catalysing the deprotonation of the pyrrole nitrogen, thus promoting the metallation reaction; this role still requires experimental evidence.

From close inspection of bacterial genomes (Table S1), we can say that the *Paracoccus* type CbiX is used throughout the α-proteobacteria for siroheme synthesis and is also used by some firmicutes and other denitrifying proteobacteria. In other organisms, including almost all archaea and sulphate reducing bacteria (that use siroheme for alternate haem biosynthesis pathway and for sulphur/nitrogen assimilation), a smaller protein has been annotated as CbiX, but their genomes also contain a gene encoding CysG (multifunctional siroheme synthase). Therefore, CbiX^S^ in these organisms may well be a cobaltochelatase as originally assumed.

The requirement for CbiX to function to synthesize siroheme for both aerobic and anaerobic assimilation of nitrite, as well as for anaerobic dissimilation of nitrite, none of which are obligatory processes for the growth of *Paracoccus* species, raises questions about the regulation of its transcription in such organisms. In *Paracoccus* the *nir* operon is regulated by NNR (*Nitrate Nitrite Reduction* transcriptional activator) but *cbiX* is found elsewhere (Fig. S2) in the genome. The upstream region of *cbiX* from *Paracoccus* also lacks the characteristic NNR binding site as evident from sequence alignments (Fig. S3). Furthermore, a transcriptomics analysis (M.J. Sullivan, G. Giannopoulos, S.J. Ferguson and D.J. Richardson, pers. comm.) has shown that the readily detectable level of transcription for *cbiX* did not alter on switching growth conditions from aerobic to anaerobic respiration on medium containing NH_4_^+^, suggesting that this chelatase is constitutively expressed and is required both during aerobic and anaerobic growth of *P. denitrificans*. These observations are consistent with the data presented in this paper, as siroheme is needed for growth on minimal medium so as to permit reduction of sulphite and nitrite for assimilation under both aerobic and anaerobic conditions, but also is required as the precursor of *d*_1_ haem, which is only required during anaerobic growth conditions for respiration.

On the other hand, when *cbiX* is present at the end of the *nir* operon, it remains to be seen if its transcription is regulated by the NNR promoter that is present upstream of *nirS* (the structural gene encoding cytochrome *cd*_1_ nitrite reductase) (Fig. S4). For example, in *Roseobacter denitrificans cbiX* overlaps (43 bp) with *nirN* gene and thus could be under NNR control, since a binding site for latter is at the 5′ end of *nir* operon. For assimilatory processes, in this organism it remains to be elucidated how any control by NNR is abrogated under aerobic growth conditions when assimilation of nitrite and sulphite is required.

## Experimental procedures

### DNA manipulations

DNA manipulations were performed by standard methods (Sambrook and Russell, 2001). Primers were synthesized by Sigma–Genosys. Amplifications by the polymerase chain reaction (PCR) used KOD DNA polymerase (from *Thermococcus kodakaraensis*) were according to supplier's instructions (Novagen). All constructs generated by PCR were confirmed as correct by sequencing.

### Cloning of *P. pantotrophus* *cbiX* and construction of *cbiX* variants

The *cbiX* ORF was amplified from *P. pantotrophus* genomic DNA using SBcbiXF and SBcbiXR, digested with NdeI and BamHI and ligated into NdeI/BamHI-digested pET14b (for over expression in *E. coli*) to obtain pSB103. Cloning in pET14b allowed the CbiX protein to be expressed with an N-terminal hexa-histidine tag. QuikChange PCRs were used to construct the desired point mutations in *cbiX* using pSB103 as template DNA. The DNA was purified with a PCR Purification Kit (Qiagen) and the template DNA was digested with DpnI. *E. coli* DH5α cells were transformed with the mutant DNA and 5 ml LB cultures were grown from the resultant transformants. After overnight growth, the plasmid DNA was extracted using a Spin Miniprep Kit (Qiagen) and the presence of the correct point mutations was validated by DNA sequencing. All the bacterial strains, primers and plasmids used in this study are shown in Table S2.

### Complementation of *E. coli* *cysG* mutant strain with *P. pantotrophus* CbiX

Plasmid pSB103 and its mutant variants were transformed into Cys247 strain that lacks the sirohydrochlorin ferrochelatase activity. It is an *E. coli* 302Δa (Brindley *et al*., 2003) derivative strain deleted in the *cysG* and harbouring plasmid pCIQ-*cobA* for expression of the *Pseudomonas denitrificans cobA* genes. The *E. coli* Cys247 strain containing pSB103 plasmid or variants were selected on LB plates supplemented with 100 μg ml^−1^ ampicillin and 34 μg ml^−1^ chloramphenicol. Strain *E. coli* 302Δ*a* was also transformed with the plasmid pKK223.2-*cysG*, which expresses the *E. coli* CysG, pET14b-*cbiK*, which expresses Se-CbiK or with an empty pET14b plasmid and grown on minimal medium plates in the absence and in the presence of cysteine ± cobalt chloride, in order to provide the positive and negative controls of the experiment respectively.

### Construction of *P. denitrificans* *cbiX* unmarked gene deletion strain

The upstream region of *cbiX* locus (∼ 900 bp) was amplified from *P. denitrificans* genomic DNA using oligonucleotides SB243 and SB244. This PCR product was gel extracted and digested with EcoRI and XbaI restriction enzymes and cloned into EcoRI/XbaI cut pTZ19R to give pSB157 construct. Similarly, the downstream region of *cbiX* locus (∼ 500 bp) was amplified from *P. denitrificans* genomic DNA using oligonucleotides SB245 and SB246 and cloned into XbaI/PstI cut pTZ19R vector to give pSB158 construct. *cbiX* upstream region was subcloned from pSB157 into EcoRI/XbaI double digested pSB158 to give pSB159. The cassette containing the 5′3′ fusion of *cbiX* flanking regions was excised from pSB159 using EcoRI and PstI enzymes, and cloned into the suicidal vector pK18mobsacB (Harighi, 2009) to generate pSB160, i.e. pK18mobsacB2-Δ*cbiX*. An unmarked deletion was generated in *cbiX* by first transferring the pSB160 into *P. denitrificans* PD1222 by conjugative mating from *E. coli* SM10 (Simon *et al*., 1983) as described previously (Moir and Ferguson, 1994).

The first recombination event was selected for using Rifampicin (for PD1222) and Kanamycin resistance (for pK18mobsacB). A second recombination event was selected for using sucrose (Fluka). Given that a second recombination can occur, resulting in either the parental genotype or the mutant genotype, correct integration of the disruption cassette was confirmed by PCR screening using primers SB243 and SB246. The resultant *cbiX* knockout strain in *P. denitrificans* is termed SBN69.

### Recombinant production and purification of CbiX and its variants

Over expression of CbiX and its variants was performed in the *E. coli* strain BL21 codonplus (RIPL) (Stratagene). All cells expressing protein were grown at 37°C in 500 ml volumes of Luria–Bertani (LB) broth in 2 l flasks from overnight starter cultures to an OD_600_ of 0.8–1.0 and transferred to 18°C before induction with 0.1 mM IPTG. After further incubation for 16 h the cells from 2 l culture were harvested and re-suspended in 25 ml of Buffer A (50 mM Tris-HCl, 200 mM NaCl, pH 7.5), containing a trace amount of DNaseI and EDTA-free protease inhibitor tablet (Roche). The total cell lysate were obtained by sonication on ice for 5 min and removing the insoluble material by centrifuging at 15 000 *g* for 40 min. The total cell lysate was applied to 5 ml of chelating sepharose fast flow column (5 ml) (GE, Healthcare), previously charged with NiCl_2_ and equilibrated with Buffer A. The column was first washed with 3 column volumes of Buffer A followed by washes with 10 column volumes of 50 mM Tris-HCl, 200 mM NaCl and 25 mM imidazole (pH 7.5), and the protein was eluted with 50 mM Tris-HCl (pH 8.0), 400 mM imidazole, according to the manufacturer's instructions. All the CbiX variants were also produced in the same manner. Purity of the samples was checked by running SDS-PAGE 10% Bis-Tris NuPAGE gels (Invitrogen).

### Structure determination and refinement

Purified CbiX was concentrated to 22 mg ml^−1^ using 10 kDa MW cut-off centrifugal concentrator devices (Millipore). Crystallization was carried out using the sitting drop vapour diffusion method at 21°C. Crystals were obtained in 0.2 M imidazole malate pH 5.5, 24% PEG 600 at a ratio of 50:50 ratio of protein : mother liquor and 10% ethylene glycol. The best crystals diffracted to 1.9 Å resolution and belonged to the space group P6_1_22. Native data were collected at the ESRF in Grenoble, beamline ID29 (λ = 0.87 Å). For phasing, crystals were soaked in a solution of mother liquor plus 5% v/v of a saturated K_2_PtCl_4_ stock solution for 5 min. Crystals were then harvested and flash frozen in the same solution as for the native crystals. Data were collected at the Diamond light source, beamline I04-1 (λ = 0.92 Å) (Table 2). PDB code for Pp-CbiX structure is 4CCS.

**Table 2:** Data collection, processing and refinement parameters of CbiX

Data collection	Native dataset	Platinum dataset
Space group	P6_1_22	P6_1_22
Cell dimensions		
*a, b, c* (Å)	130.45, 130.45, 56.85	129.99, 129.99, 56.34
*α, β, ϒ* (°)	90.0, 90.0, 120.0	90.0, 90.0, 120.0
Wavelength	0.87	0.917
Resolution (Å)	65.2–1.9 (1.95–1.9)	56.2–2.7 (2.8–2.7)
*R*_sym_ or R_merge_	0.078 (0.751)	0.094 (0.962)
*I*/σ*I*	24.9 (3.7)	30.1 (6.5)
Completeness (%)	100.0 (100.0)	99.8 (99.2)
Redundancy	14.2 (14.6)	37.0 (34.2)
Refinement		
Resolution (Å)	65.2–1.9	
No. of reflections	22942	
*R*_work_/*R*_free_	19.4/23.7	
No of atoms		
Protein	1674	
Ligand/ion	253	
Water	239	
B-factors		
Protein	31.8	
Ligand/ion		
Water		
RMSD		
Bond lengths (Å)	0.01	

Data were processed using xia2 (Winter, 2010), and autoSHARP (Vonrhein *et al*., 2007) used to identify the first Platinum site. The hand was determined at this point by SOLOMON (Abrahams and Leslie, 1996) solvent flattening, showing the original hand is the correct one (CC for observed verses solvent flattened Es was 17.5% versus 11.7%). Inspection of SHARP LLG (Log Likelihood gradient) (Bricogne *et al*., 2003) maps then identified a further two Platinum sites. Solvent content was optimized to 52.8%.

Buccaneer (Cowtan, 2006) was used to build into the initial solvent flattened map, followed by manual refinement in COOT (Emsley *et al*., 2010) and autoBUSTER (Bricogne *et al*., 2011). The final model is a continuous stretch from residues 3–228. There is one protomer of CbiX in the asymmetric unit, along with one ordered malic acid and one ethylene glycol molecule. The final *R*/*R*_free_ values are 19.4%/23.7%. 99.55% of the residues lie in the favoured region of the Ramachandran plot, and there are no outliers. The structure has a MolProbity (Chen *et al*., 2010) score of 0.98, making the structure in the 100th percentile for all structures in the resolution range 1.90 Å + 0.25 Å.

## References

[b1] Abrahams JP, Leslie AGW (1996). Methods used in the structure determination of bovine mitochondrial F1 ATPase. Acta Crystallogr D Biol Crystallogr.

[b2] Bali S, Lawrence AD, Lobo SA, Saraiva LM, Golding BT, Palmer DJ (2011). Molecular hijacking of siroheme for the synthesis of heme and *d*_1_ heme. Proc Natl Acad Sci USA.

[b3] Bricogne G, Vonrhein C, Flensburg C, Schiltz M, Paciorek W (2003). Generation, representation and flow of phase information in structure determination: recent developments in and around SHARP 2.0. Acta Crystallogr D Biol Crystallogr.

[b1001] Bricogne G, Blanc E, Brandl M, Flensburg C, Keller P, Paciorek W (2011).

[b5] Brindley AA, Raux E, Leech HK, Schubert HL, Warren MJ (2003). A story of chelatase evolution – identification and characterization of a small 13–15-kda ‘ancestral’ cobaltochelatase (CbiX^S^) in the archaea. J Biol Chem.

[b6] Chen VB, Arendall WB, Headd JJ, Keedy DA, Immormino RM, Kapral GJ (2010). MolProbity: all-atom structure validation for macromolecular crystallography. Acta Crystallogr D Biol Crystallogr.

[b7] Cowtan K (2006). The buccaneer software for automated model building. 1. Tracing protein chains. Acta Crystallogr D Biol Crystallogr.

[b8] Emsley P, Lohkamp B, Scott WG, Cowtan K (2010). Features and development of Coot. Acta Crystallogr D Biol Crystallogr.

[b9] Fischer M, Schmidt C, Falke D, Sawers RG (2012). Terminal reduction reactions of nitrate and sulphate assimilation in *Streptomyces coelicolor A3(2)*: identification of genes encoding nitrite and sulphite reductases. Res Microbiol.

[b10] Hansen J, Muldbjerg M, Cherest H, SurdinKerjan Y (1997). Siroheme biosynthesis in *Saccharomyces cerevisiae* requires the products of both the MET1 and MET8 genes. FEBS Lett.

[b1003] Hansson M, Hederstedt L (1994). Purification and characterisation of a water-soluble ferrochelatase from *Bacillus subtilis*. Eur J Biochem.

[b1004] Hansson MD, Karlberg T, Rahardja MA, Al-Karadaghi S, Hansson M (2007). Amino acid residues His183 and Glu264 in *Bacillus subtilis* ferrochelatase direct and facilitate the insertion of metal ion into protoporphyrin IX. Biochemistry.

[b1005] Hansson MD, Karlberg T, Söderberg CA, Rajan S, Warren MJ, Al-Karadaghi S (2011). Bacterial ferrochelatase turns human: Tyr13 determines the apparent metal specificity of *Bacillus subtilis* ferrochelatase. J Biol Inorg Chem.

[b11] Harighi B (2009). Genetic evidence for *CheB*- and *CheR*-dependent chemotaxis system in *A. tumefaciens* toward acetosyringone. Microbiol Res.

[b12] Leech HK, Raux-Deery E, Heathcote P, Warren MJ (2002). Production of cobalamin and sirohaem in *Bacillus megaterium*: an investigation into the role of the branchpoint chelatases sirohydrochlorin ferrochelatase (SirB) and sirohydrochlorin cobalt chelatase (CbiX). Biochem Soc Trans.

[b13] Moir JWB, Ferguson SJ (1994). Properties of a *Paracoccus denitrificans* mutant deleted in cytochrome *c*_550_ indicate that a copper protein can substitute for this cytochrome in electron-transport to nitrite, nitric-oxide and nitrous-oxide. Microbiology.

[b14] Moore SJ, Warren MJ (2012). The anaerobic biosynthesis of vitamin B_12_. Biochem Soc Trans.

[b15] Raux E, Thermes C, Heathcote P, Rambach A, Warren MJ (1997). A role for *Salmonella typhimurium cbiK* in cobalamin (Vitamin B_12_) and siroheme biosynthesis. J Bacteriol.

[b16] Raux E, McVeigh T, Peters SE, Leustek T, Warren MJ (1999). The role of *Saccharomyces cerevisiae* Met1p and Met8p in sirohaem and cobalamin biosynthesis. Biochem J.

[b17] Raux E, Leech HK, Beck R, Schubert HL, Santander PJ, Roessner CA (2003). Identification and functional analysis of enzymes required for precorrin-2 dehydrogenation and metal ion insertion in the biosynthesis of sirohaem and cobalamin in *Bacillus megaterium*. Biochem J.

[b18] Romao CV, Ladakis D, Lobo SAL, Carrondo MA, Brindley AA, Deery E (2011). Evolution in a family of chelatases facilitated by the introduction of active site asymmetry and protein oligomerization. Proc Natl Acad Sci USA.

[b19] Sambrook J, Russell DW (2001). Molecular Cloning: A Laboratory Manual.

[b20] Schubert HL, Rose RS, Leech HK, Brindley AA, Hill CR, Rigby SEJ, Warren MJ (2008). Structure and function of SirC from *Bacillus megaterium*: a metal-binding precorrin-2 dehydrogenase. Biochem J.

[b21] Shearer N, Hinsley AP, Van Spanning RJM, Spiro S (1999). Anaerobic growth of *Paracoccus denitrificans* requires cobalamin: characterization of *cobK* and *cobJ* genes. J Bacteriol.

[b1002] Simon J, Kroneck PMH (2013). Microbial sulfite respiration. Adv Microb Physiol.

[b22] Simon R, Priefer U, Puhler A (1983). A broad host range mobilization system for *in vivo* genetic engineering – Transposon mutagenesis in gram negative bacteria. Nat Biotechnol.

[b23] Storbeck S, Walther J, Mueller J, Parmar V, Schiebel HM, Kemken D (2009). The *Pseudomonas aeruginosa nirE* gene encodes the S-adenosyl-L-methionine-dependent uroporphyrinogen III methyltransferase required for heme *d*_1_ biosynthesis. FEBS J.

[b24] Storbeck S, Saha S, Krausze J, Klink BU, Heinz DW, Layer G (2011). Crystal structure of the heme *d*_1_ biosynthesis enzyme NirE in complex with its substrate reveals new insights into the catalytic mechanism of S-Adenosyl-L-methionine-dependent uroporphyrinogen III Methyltransferases. J Biol Chem.

[b25] Stroupe ME, Leech HK, Daniels DS, Warren MJ, Getzoff ED (2003). CysG structure reveals tetrapyrrole-binding features and novel regulation of siroheme biosynthesis. Nat Struct Biol.

[b26] Swamy U, Wang MT, Tripathy JN, Kim SK, Hirasawa M, Knaff DB, Allen JP (2005). Structure of spinach nitrite reductase: implications for multi-electron reactions by the iron-sulfur: siroheme cofactor. Biochemistry.

[b27] Tripathy BC, Sherameti I, Oelmuller R (2010). Siroheme: an essential component for life on earth. Plant Signal Behav.

[b28] Vonrhein C, Blanc E, Roversi P, Bricogne G, Doublié S (2007). Automated structure solution with autoSHARP. Macromol Cryst Protocols.

[b29] Warren MJ, Bolt EL, Roessner CA, Scott AI, Spencer JB, Woodcock SC (1994). Gene dissection demonstrates that the *Escherichia coli cysG* gene encodes a multifunctional protein. Biochem J.

[b30] Winter G (2010). xia2: an expert system for macromolecular crystallography data reduction. J Appl Cryst.

[b31] Yin J, Xu LX, Cherney MM, Raux-Deery E, Bindley AA, Savchenko A (2006). Crystal structure of the *vitamin B*_12_ biosynthetic cobaltochelatase, CbiX^S^, from *Archaeoglobus fulgidus*. J Struct Funct Genomics.

[b32] Zajicek RS, Bali S, Arnold S, Brindley AA, Warren MJ, Ferguson SJ (2009). *d*_1_ haem biogenesis – assessing the roles of three *nir* gene products. FEBS J.

