# Supplementary material for: Identification and characterization of the ‘missing’ terminal enzyme for siroheme biosynthesis in α-proteobacteria
Source: Mol Microbiol. 2014 Mar 13;92(1):153–63. doi: 10.1111/mmi.12542 (PMC4063343; doi:10.1111/mmi.12542)
Supplement: Supplementary file 1 [file mmi0092-0153-SD1.pdf]

SUPPORTING INFORMATION

Identification and Characterisation of the 'missing' Terminal Enzyme for Siroheme Biosynthesis in α-Proteobacteria

Shilpa Bali[1], Sarah Rollauer[2], Pietro Roversi[3], Evelyne Raux-Deery[4], Susan M. Lea[2], Martin J. Warren[4] and Stuart J. Ferguson[1]

Address correspondence to**: Stuart J. Ferguson,** stuart.ferguson@bioch.ox.ac.uk

Correspondence to: Tel.: 01865613299; Fax: 01865613201

1. Department of Biochemistry, University of Oxford, South Parks Road, OX1 3QU, UK. 2. Sir William Dunn School of Pathology, University of Oxford, South Parks Road, Oxford OX1 3RE, UK. 3. Global Phasing Ltd. Sheraton House, Castle Park, Cambridge CB3 0AX, UK. 4. School of Biosciences, University of Kent, Canterbury, Kent, CT2 7NJ, United Kingdom

Key Words: Ferrochelatase, *Paracoccus pantotrophus*, Siroheme, Structure, Anaerobic biosynthesis and Tetrapyrrole.

FigureS1

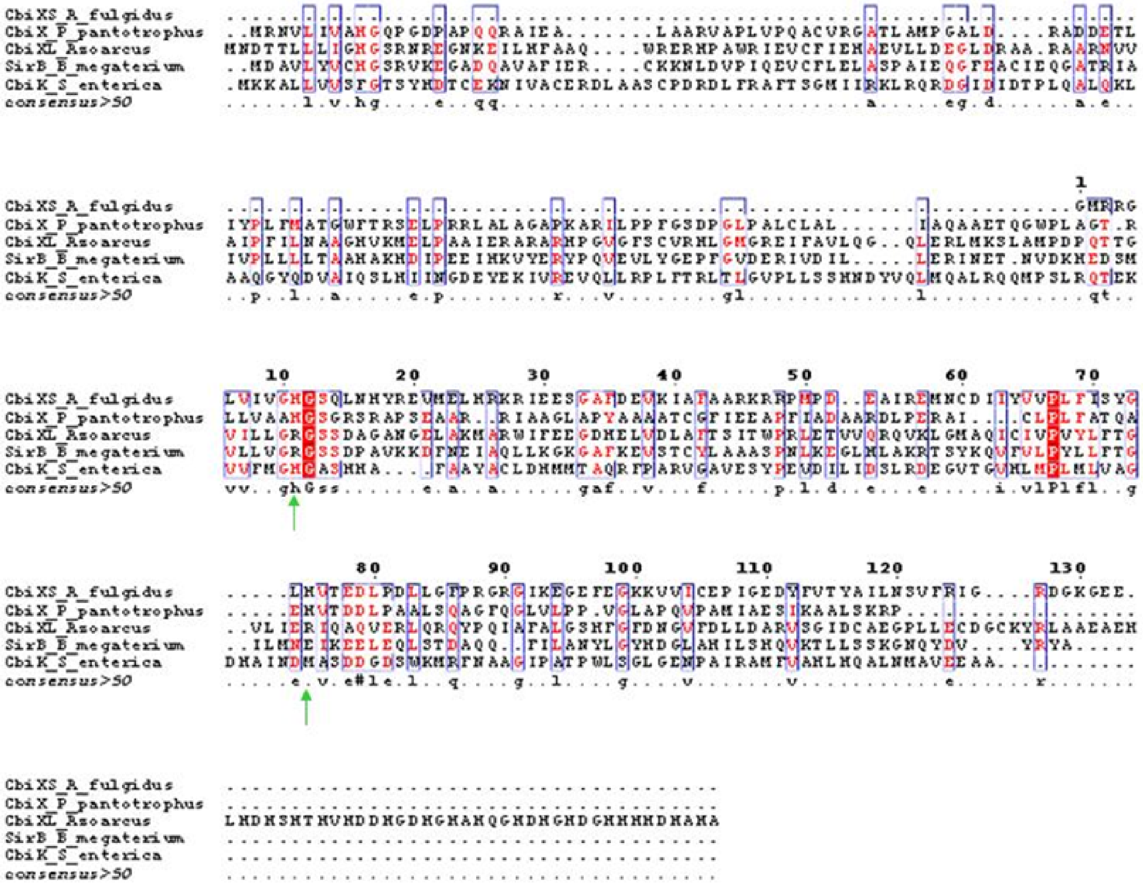

Fig.S1: Multiple sequence alignment of different type II chelatases with CbiX from *P. pantotrophus.* Sequence alignment shows the two histidines (His127 and His187), from *P. pantrotrophus*-CbiX that are important for the ferrochelatase function; they are pointed by the green arrows.

Figure S2

Organism Paracoccus denitrificans PD1222 (strain: PD1222)

Lineage Bacteria; Proteobacteria; Alphaproteobacteria; Rhodobacterales; Rhodobacteraceae; Paracoccus

Genomic context

Location: chromosome: 1

Sequence: Chromosome: 1; NC_008686.1 (2330519..2331211, complement)

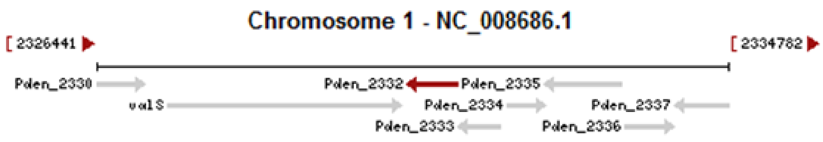

ORF number in PD1222	Functional Annotation
Pdne_2331	Valyl-tRNA synthetase
Pdne_2332	CbiX or SirB like chelatase
Pdne_2333	Glutamyl-tRNA synthetase
Pdne_2334	NnrU family of Protein
Pdne_2335	FAD-dependent oxidoreductase
Pdne_2336	Oxidoreductase has AdoMet domain

Fig.S2: Genetic context of *P. denitrificans.* Gene highlighted in red arrow (Pdne_2332) is annotated as *cbiX* (putative cobalt chelatase) is shown to be a ferrochelatase of sirohydrochlorin in *Paracoccus* genus by *in vivo* functional analysis. It is found next to Pdne_2333 that annotates as glutamyl-tRNA synthetase and has a role in 5-aminolevulinic acid (ALA) synthesis that is a universal tetrapyrrole precursor. In some denitrifiers, such as *Roseobacter denitrificans*, this *cbiX* gene is found right next to *nirN* of d_1 heme biosynthesis operon (refer to FigS4).

Figure S3

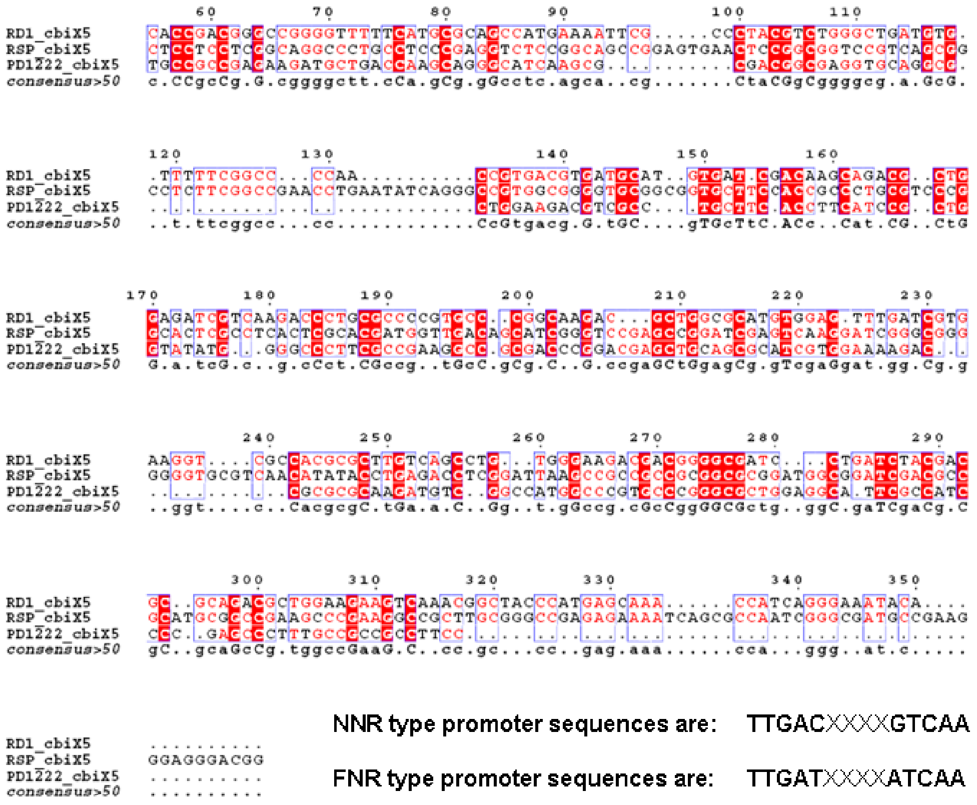

Fig.S3: Multiple sequence alignment of the upstream region of *cbiX* gene from *some denitrifiers.* Sequence alignment of the 5' flanking region of *cbiX* gene from *P. denitrificans* (PD1222), *Roseobacter denitrificans* (RD1) and *Rhodobacter sphaeroids* (RSP) is compared for the presence of NNR and FNR type promoter sequence consensus. Alignment from this region clearly shows the lack of these type of promoter sequences.

Figure S4

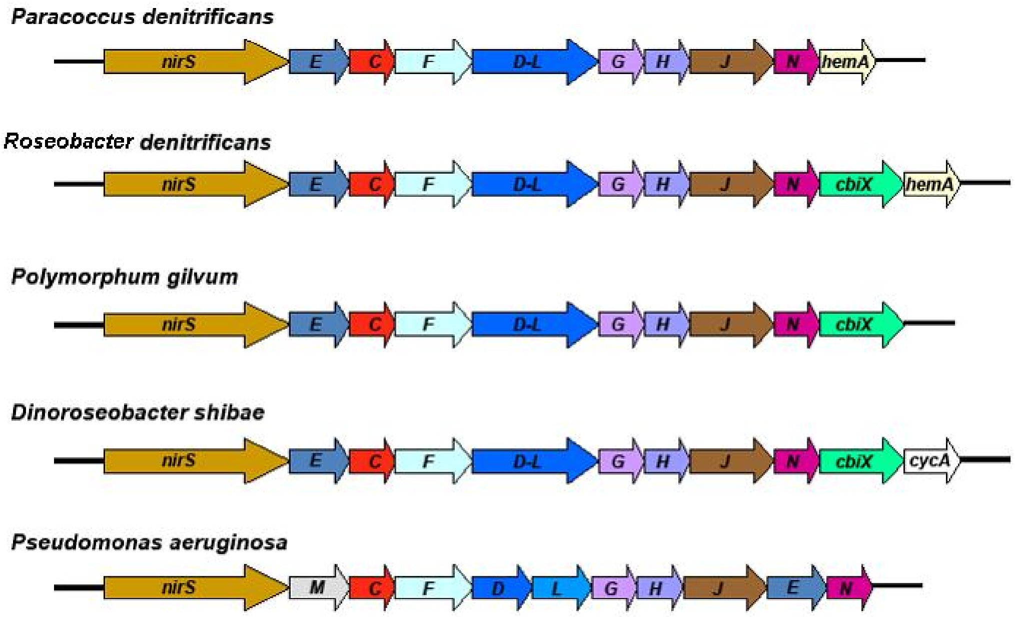

Fig.S4: Genetic organisation of the *nirS* loci in different denitrifying bacteria. Clusters were generated from the "Microbial Genome Database for Comparative Analysis" (http://mbgd.genome.ad.jp.

Figure S5

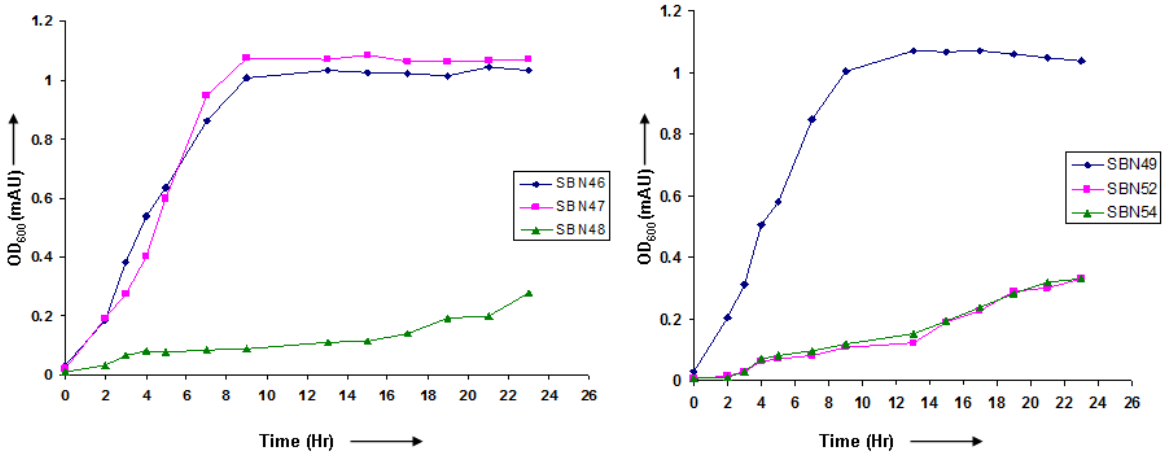

Fig.S5: Growth curves for *E. coli* (Cys247) variant strains. Representative strains from Table 1 were grown aerobically on minimal medium supplemented with ampicillin and 0.1 g/L yeast extract at 37 °C. Both SBN52 (Cys247::Pp-CbiX^{H127A}) and SBN54 (Cys247::Pp-CbiX^{H187A}), when supplemented with 20 mg/L of L-cysteine, could grow to same levels as SBN49 (Cys247::WT Pp-CbiX). SBN46 (Cys247::*E. coli*-CysG) and SBN47 (Cys247::Se-CbiK) are the positive controls, and SBN48 (Cys247::Expression vector only) represents the negative control for the growth experiments.

Table S1: List of denitrifying bacteria and few archaea that express dissimilatory cytochrome cd_1 nitrite reductase (NirS).

	Denitrifying bacteria	NirS	Siroheme	CbiX<br>Ferrochelatase	CysG<br>Siroheme synthase
1	Roseobacter denitrificans	Yes	Yes	Yes	No
2	Paracoccus pantotrophus	Yes	Yes	Yes	No
3	Paracoccus denitrificans	Yes	Yes	Yes	No
4	Rhodobacter sphaeroieds	Yes	Yes	Yes	No
5	Polymorphum gilvum	Yes	Yes	Yes	No
6	Ruegeria pomeroyi	Yes	Yes	Yes	No
7	Dinoroseobacter shibae	Yes	Yes	Yes	No
8	Phaeobacter gallaeciensis	Yes	Yes	Yes	No
9	Pseudovibrio species	Yes	Yes	Yes	No
10	Magnetosipirillum gry MSR1	Yes	Yes	Yes	No
11	Magnetospirillum magneticum AMB-1	Yes	Yes	Yes	No
12	Deinococcus species	Yes	Yes	Yes	No
13	Mycobacterium abscessus	Yes	Yes	Yes	No
14	Bradyrhizobium oligotrophicum	Yes	Yes	Yes	No
15	Pseudomonas species	Yes	Yes	—	Yes
16	Ps. stutzeri	Yes	Yes	—	Yes

17	Ps. denitrificans	Yes	Yes	—	Yes
18	Ps. aeruginosa	Yes	Yes	—	Yes
19	Ps. syringae	Yes	Yes	—	Yes
20	Hahella chejuensis KCTC 2396	Yes	Yes	—	Yes
21	Sulfuricella denitrificans skB26	Yes	Yes	—	Yes
22	Thiobacillus denitrificans ATCC 25259	Yes	Yes	—	Yes
23	Beggiatoa sp. PS	Yes	Yes	—	Yes
24	Pseudogulbenkiania sp. NH8B	Yes	Yes	—	Yes
25	Sideroxydans lithotrophicus ES-1	Yes	Yes	—	Yes
26	gamma proteobacterium HdN1	Yes	Yes	—	Yes
27	Cupriavidus taiwanensis	Yes	Yes	Yes like $CbiX^S$	No
28	Dechloromonas aromatica RCB	Yes	Yes	Yes	No
29	Rubrivivax benzoatilyticus	Yes	Yes	Yes like SirB	No
30	Rubrivivax gelatinosus IL144	Yes	Yes	Yes like SirB	No
31	Marinobacter hydrocarbonoclasticus ATCC 49840	Yes	Yes	Yes like $CbiX^S$	No
32	Aromatoleum aromaticum EbN1	Yes	Yes	Yes like SirB	No
	**Aerobic archaea**	**NirS**	**Siroheme**	**$CbiX^S$ cobaltchelatase**	**CysG Siroheme**

					**synthase**
33	Pyrobaculum aerophilum IM2	Yes	Yes	Yes	Yes
34	Pyrobaculum arsenaticum DSM13514	Yes	Yes	Yes	Yes
35	Pyrobaculum calindifontis JCM11548	Yes	Yes	Yes	Yes

Table S2: Bacterial strains, Plasmids and Primers used in this study.

**Strain *E. coli***	**Genotype**	**Reference**
DH5α	F⁻ φ80d*lac*ZΔM15, *recA*1, *endA*1, *gyr*AB, *thi*-1, *hsdR*17(r_K-, m_K+), *supE*44, *relA*1, *deo*R, Δ*(lacZYA-arg*F) U169, *pho*A	Invitrogen
BL21-CodonPlus (DE3)-RIPL	F⁻, *omp*T, *hsd*S_B(r_B-, m_B-), *dcm*, *gal*, λ(DE3), *endA*1, Hte (*argU, proL*, Cam^r)	Stratagene
CysG 247 or (302Δa::pCIQ-*cobA*)	*E. coli* 302Δa derivative strain deleted in the *cysG* and harboring plasmid pCIQ-*cobA* for expression of the *Pseudomonas denitrificans cobA* gene	Brindley et. al., 2003
Pd1222	*P. denitrificans, wt* strain	Moir et. al., 1994
SBN69	*P. denitrificans ΔcbiX* strain	This work
SBN70	*P. denitrificans ΔcbiX* strain with *cbiX* on plasmid.	This work
**Primer Name**	**Sequence**	**Plasmid**
SBcbiXF	AAAACATATGGTGCATAAGGTGCTGGTCGCCCATGGCCAG	pSB103
SBcbiXR	AAAAGGATCCTCAGGGCCGCTTGGGAAGGGCGGCCTTGATGCTTTC	
SB215	ACGTCCTCATCGTCGCCGCGGGCCAGCCCGGCG	pSB132
SB216	CGCCGGGCTGGCCCGCGGCGACGATGAGGACGTT	
SB217	CATCGTCGCCCATGGCGCACCCGGCGACCCCGC	pSB133
SB218	GCGGGGTCGCCGGGTGCGCCATGGGCGACGATG	

SB219	GCTGCTGGTCGCCGCCGCCGGCTCCGGCCGCTCGC	pSB134
SB220	CGAGCGGCCGGAGCCGGCGGCGGCGACCAGCAGC	
SB221	GCCGAGCACGTCACCGCCGACCTGCCCGCGGCGC	pSB135
SB222	GCGCCGCGGGCAGGTCGGCGGTGACGTGCTCGGC	
SB223	GCCACCCAGGCCGAGCACGTCACCGACGACCTGCG	pSB136
SB224	CAGCAGGTCGTCGGTGACGTGCTCGGCCTGGGTGG	
SB233	GCCGAGCACGTCACCGACGCCCTGCCCGCGGCGC	pSB140
SB234	GCGCCGCGGGCAGGGCGTCGGTGACGTGCTCGGC	
SB235	GCCACCCAGGCCGCGCACGTCACCGACGACCTGC	pSB141
SB236	GCAGGTCGTCGGTGACGTGCGCGGCCTGGGTGGC	
SB243	TCGTCAGAATTCTCTGGAACGCCACCCGCTTTGCCG AGATGAACGGCGTT	pSB157
SB244	AGCGGCTCTAGACTTCTCGTTCTCCAAATACTCATGC GACCGCGCCAT	
SB245	CACCTTTCTAGAGGAAAGCGGCGGAGAAGGGTTCG GGGATGGCGCATTCCTCCA	pSB158
SB246	GCCAACTGCAGTTCGCCCCATTCCATGCCGAAAGCC TAGCCTTGCGCCCGTGC	
**Plasmid**	**Description**	**Reference**
pSB103	Wild-type, *cbiX* in pET14b	This work
pSB131	Wild-type, *cbiX* with Strep Tag in pEG276	This work
pSB132	pSB103 *cbiX*^{H10A}	This work
pSB133	pSB103 *cbiX*^{Q12A}	This work
pSB134	pSB103 *cbiX*^{H127A}	This work
pSB135	pSB103 *cbiX*^{D190A}	This work
pSB136	pSB103 *cbiX*^{H187A}	This work
pSB140	pSB103 *cbiX*^{D191A}	This work
pSB141	pSB103 *cbiX*^{E186A}	This work
pSB157	*cbiX*^{UpperFlank} in pTZ19R	This work
pSB158	*cbiX*^{DownFlank} in pTZ19R	This work
pSB159	*cbiX*$^{Upper+DownFlank}$ in pTZ19R	This work
pSB160	*cbiX*$^{Upper+DownFlank}$ in pK18mobsacB or pK18mobsacB2-Δ*cbiX*	This work
**Cys247 derivative strains**	**Description**	**Reference**
SBN46	Cys247::pET14b-*cysG_Ecoli*	This work

SBN47	Cys247::pET14b-*cbiK_Se*	This work
SBN48	Cys247::pET14b	This work
SBN49	Cys247::pET14b-*cbiX_Pp*	This work
SBN50	Cys247::pET14b-*cbiX*^{H10A}	This work
SBN51	Cys247::pET14b-*cbiX*^{Q12A}	This work
SBN52	Cys247::pET14b-*cbiX*^{H127A}	This work
SBN53	Cys247::pET14b-*cbiX*^{D190A}	This work
SBN54	Cys247::pET14b-*cbiX*^{H187A}	This work
SBN67	Cys247::pET14b-*cbiX*^{D191A}	This work
SBN68	Cys247::pET14b-*cbiX*^{E186A}	This work
